# Supplementary figures and images for: A single-molecule method for measuring fluorophore labeling yields for the study of membrane protein oligomerization in membranes
Source: PLoS One. 2023 Jan 20;18(1):e0280693. doi: 10.1371/journal.pone.0280693 (PMC9858377; doi:10.1371/journal.pone.0280693)

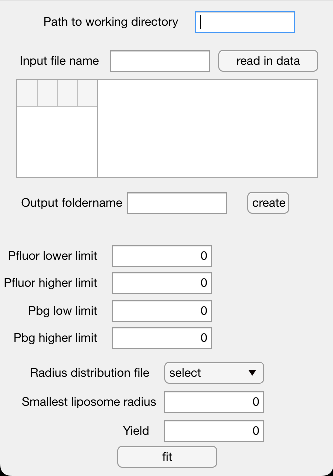

Supplement: S1 File — This program takes the liposome size distribution, the reconstitution yield, the smallest liposome size radius that allows for occupancy of the liposome by two subunits, the experimental dimer photobleaching distribution and the (Pfluor, Pbg) range as inputs. It then generates raw SSR values of the experimental data vs. the model while iterating over (Pfluor, Pbg) and further carries out the peak determination from Norm. SSR-1 and the variance analysis on bootstrapping of PSSR. This MATLAB application was written using the Mac version of MATLAB (R2020b). Previous versions may not support the application and different operating systems might need adjustments to the code. The following toolbox needs to be installed for running the app: "Statistics and Machine Learning Toolbox". A detailed step-by-step instruction of using the app can be found at: https://github.com/tnozturk/smPBfit. (MLAPP) [file pone.0280693.s001.mlapp › metadata/appScreenshot.png]

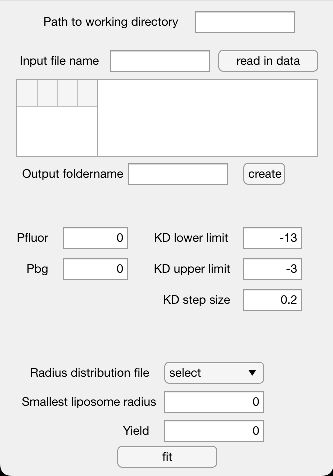

Supplement: S2 File — This program takes the liposome size distribution, the experimental photobleaching distribution, the designated (Pfluor, Pbg) values, the reconstitution yield, the smallest liposome size radius that allows for occupancy of the liposome by two subunits, and the desired KD range and step size as inputs. It then generates the raw SSR values of the experimental data vs. the model while iterating over KD and further carries out the peak determination from Norm. SSR-1 and the variance analysis on bootstrapping of PSSR. This MATLAB application was designed using the Mac version of MATLAB (R2020b). Previous versions might not support the application and different operating systems might need adjustments to the code. The following toolbox needs to be installed for running the app: "Statistics and Machine Learning Toolbox". A detailed step-by-step instruction of using the app can be found at: https://github.com/tnozturk/smPBfit. (MLAPP) [file pone.0280693.s002.mlapp › metadata/appScreenshot.png]
